# Towards noninvasive estimation of tumour pressure by utilising MR elastography and nonlinear biomechanical models: a simulation and phantom study

**DOI:** 10.1038/s41598-020-62367-3

**Published:** 2020-03-27

**Authors:** Daniel Fovargue, Marco Fiorito, Adela Capilnasiu, David Nordsletten, Jack Lee, Ralph Sinkus

**Affiliations:** 1 School of Biomedical Engineering and Imaging Sciences, King’s College London, London, United Kingdom; 2 Department of Biomedical Engineering and Cardiac Surgery, University of Michigan, Ann Arbor, Michigan USA; 3 INSERM UMRS1148 - Laboratory for Vascular Translational Science, University Paris, Paris, France

**Keywords:** Cancer imaging, Tumour biomarkers, Diagnostic markers, Experimental models of disease, Translational research

## Abstract

The solid and fluid pressures of tumours are often elevated relative to surrounding tissue. This increased pressure is known to correlate with decreased treatment efficacy and potentially with tumour aggressiveness and therefore, accurate noninvasive estimates of tumour pressure would be of great value. We present a proof-of-concept method to infer the total tumour pressure, that is the sum of the fluid and solid parts, by examining stiffness in the peritumoural tissue with MR elastography and utilising nonlinear biomechanical models. The pressure from the tumour deforms the surrounding tissue leading to changes in stiffness. Understanding and accounting for these biases in stiffness has the potential to enable estimation of total tumour pressure. Simulations are used to validate the method with varying pressure levels, tumour shape, tumour size, and noise levels. Results show excellent matching in low noise cases and still correlate well with higher noise. Percent error remains near or below 10% for higher pressures in all noise level cases. Reconstructed pressures were also calculated from experiments with a catheter balloon embedded in a plastisol phantom at multiple inflation levels. Here the reconstructed pressures generally match the increases in pressure measured during the experiments. Percent errors between average reconstructed and measured pressures at four inflation states are 17.9%, 52%, 23.2%, and 0.9%. Future work will apply this method to *in vivo* data, potentially providing an important biomarker for cancer diagnosis and treatment.

## Introduction

Tumours often exhibit both increased fluid and solid pressure relative to surrounding tissue^1,2^. Increased interstitial fluid pressure (IFP) is caused by factors such as vessel leakiness and interstitial fibrosis which lead to a buildup of fluid and reduced drainage^1,3^. Elevated solid pressure originates from the proliferation of cancer cells and growth of the tumour^4^. Both increased IFP and solid pressure lead to decreased efficacy of treatment and there is some evidence that these correlate with poor prognosis, due to promotion of tumour progression and increased invasion^1–5^. IFP is able to be measured invasively by wick-in-needle^1^ and the solid pressure of tumours has been measured after extraction^2^, but clearly noninvasive measurement of IFP and/or solid pressure would be of immense value.

Such an approach is proposed here, using measurements of material properties in peritumoural tissue to infer tumour pressure. Increased fluid and solid pressure lead to the tumour pushing on surrounding tissue, which has been shown by Nia *et al*.^2^ by observing the expansion of excised tumours. In many instances this force will cause nearby tissue to deform. For example, a nearly spherical and well separated tumour exhibiting high pressure would compress nearby tissue radially and stretch it circumferentially. As tissue exhibits nonlinear response to strain, these deformations will lead to significant changes in the stiffness of the tissue near the tumour. The magnitude of these changes in stiffness will then scale with the total pressure of the tumour (the sum of fluid and solid parts) and so measurement of these stiffness changes represents a pathway towards inferring the tumour pressure noninvasively.

To measure tissue stiffness in the peritumoural tissue, magnetic resonance elastography (MRE) is used. MRE is an established magnetic resonance imaging (MRI) based method to estimate tissue stiffness based on imaging the propagation of shear waves^6–8^. Typically, the patient is subjected to single frequency vibrational waves from an external transducer while in an MR scanner. Through phase-contrast imaging, images of the wave motion are constructed. A stiffness reconstruction method is then responsible for solving an inverse problem using these wave images to create an image of the tissue stiffness (most often the shear modulus)^9^. Recent work has introduced an MRE reconstruction method that accounts for stiffness bias due to large deformation^10^. This latter approach is extended here to account for and utilise the effect of the tissue deformation due to tumour pressure. The stiffness in the vicinity of the tumour is reconstructed while incorporating radial scalings of an assumed deformation field. The scaling which most reduces the presumed biases is chosen as the correct scaling. Finally, an analytic formula based on the nonlinear material law relates this scaling to an overall tumour pressure. The method is herein applied to both simulation and phantom data.

Some previous research has investigated noninvasive approaches to tumour pressure measurement within the MRI and elastography domains. Early work showed that T1 and T2 relaxation times do not correlate with IFP measured by wick-in-needle^11^. Another approach used by several works is dynamic contrast enhanced MRI where fluid flow velocity at the tumour surface is estimated by examining contrast agent over time^12–14^. This velocity can then be used to estimate IFP by assuming or estimating hydraulic conductivity and related parameters^15,16^. Alternatively, this velocity can be used as a stand-in for IFP as strong correlation has been shown in some cases^17^ and recent work showed it may be a prognostic factor in cervical cancer^18^. An MR elastography method was published that reconstructs images of fluid pressure and hydraulic conductivity distributions in addition to tissue stiffness^19^; however, results were limited to a single numerical simulation and high errors were reported. Ultrasound elastography techniques have recently been applied to solid stress and tumours, however these have mainly focused on an analysis of the stress or stiffness inside the tumour^20–23^. In contrast to the above methods, the method presented here estimates a related but different parameter - total tumour pressure - by analysing stiffness in the tissue surrounding the tumour.

## Methods

The goal of the method presented here is to infer total tumour pressure by examining the stiffness of the tissue surrounding the tumour. There are three main components making up this method: (1) a stiffness reconstruction that accounts for nonlinear stiffness bias due to deformation, (2) a method for determining the correct scaling of the deformation prescribed to the tumour, and (3) a conversion between the deformation scaling parameter and pressure. These three components are presented in turn in this section, followed by a discussion of the simulations and phantom experiments used to validate the method.

### Stiffness reconstruction

Various approaches to elastography and stiffness (shear modulus) reconstruction have been researched including work on differing material assumptions^7–9,24–26^, however many reconstruction methods, including the method^27^ extended here assume isotropy and incompressibility. This results in the following linear time-harmonic viscoelastic equations governing the wave behaviour,

1 $$\rho {\omega }^{2}{\boldsymbol{v}}+\nabla \cdot [[{G}^{\ast }(\nabla {\boldsymbol{v}}+{(\nabla {\boldsymbol{v}})}^{T})]+\nabla p={\bf{0}}$$

2 $$\nabla \cdot {\boldsymbol{v}}=0$$

where ***v*** are the complex-valued wave displacements, *ρ* is the tissue density (assumed to be constant and equal to that of water), *ω* is the angular frequency of the transducer, and *p* is the complex hydrostatic pressure. The complex-valued shear modulus is denoted ${G}^{\ast }=G{\rm{{\prime} }}+iG{\rm{{\prime} }}{\rm{{\prime} }}$, where $G{\rm{{\prime} }}$ is the *storage modulus* or *elasticity* and *G″* is the *loss modulus* or *viscosity*. The complex-valued steady-state displacements, ***v***, and pressure, *p*, are related to their real-valued time-dependent counterparts by $\bar{{\boldsymbol{v}}}\left({\boldsymbol{x}},t\right)={\rm{Re}}\left({\boldsymbol{v}}\left({\boldsymbol{x}}\right){e}^{i\omega t}\right)$ and $\bar{p}\left({\boldsymbol{x}},t\right)={\rm{Re}}\left(p\left({\boldsymbol{x}}\right){e}^{i\omega t}\right)$. A typical MRE reconstruction will solve Eq. (1), in some manner, for *G*^∗^. Briefly, the method here computes a value for *G*^∗^ at every voxel of the wave image data using a moving local finite element mesh and assuming *G*^∗^ is locally homogeneous.

Unlike the linear wave theory utilised above, tissue is generally considered to have a nonlinear response to large deformation^10,28–32^. Subject to such deformation, stiffness can rarely be thought of as a single parameter. Most generally, stiffness is represented by the 4th order elasticity tensor relating the 2nd order stress and strain tensors. While in many cases stiffness can be reduced to fewer parameters, large deformation of isotropic tissue will result in at least some apparent anisotropy in stiffness as, for example, compression (and therefore softening) in one direction will lead to tension (and therefore hardening) in another direction. As described in Capilnasiu *et al*.^10^, the shear waves will generally observe the stiffness component corresponding to the propagation direction (as MRE uses steady state vibrational waves this is better understood as the ***k*** vector direction). So in a simple case of orthogonal deformation and wave propagation the interaction may be well understood. In complex scenarios, however, the resulting stiffness values are much harder to interpret. If the standard MRE reconstruction mentioned above is applied in these cases, then biased results are expected due to the waves observing a combination of multiple stiffness components. These complexities, however, can be fully accounted for if the biorheology of the tissue is known.

**Figure 1:**
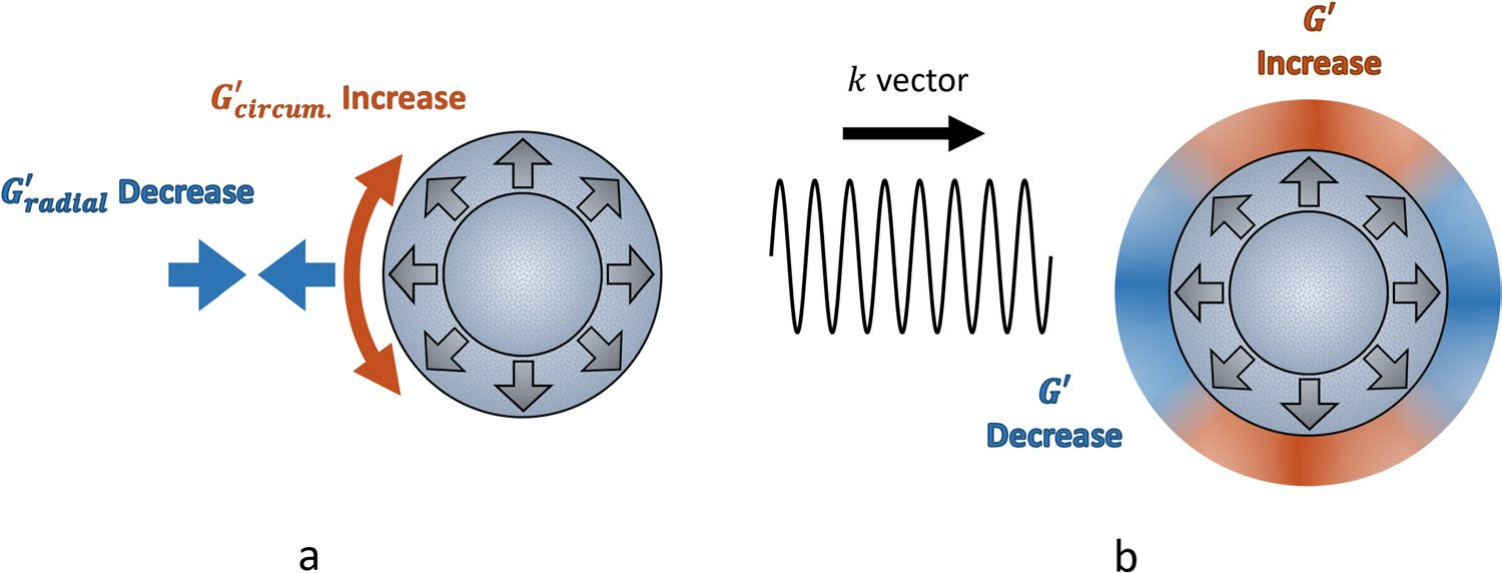
Illustration of stiffness changes near a pressurized spherical inclusion in the idealized case of shear wave propagation in a single uniform direction. (**a**) The fluid and solid pressure of the tumour causes deformation of the surrounding tissue, equivalent to the tumour inflating from some smaller size. This deformation leads to anisotropic stiffness, specifically increased circumferential stiffness and decreased radial stiffness. (**b**) The bias in the stiffness pattern reconstructed from a uniform shear wave travelling left to right. In reality this is a 3D effect, so the stiffness increase would resemble a ring-like structure around the inclusion in the transverse direction to the wave propagation.

The main model considered here for a pressurized tumour is an inflating sphere. As a spherical or some approximately spherically shaped inclusion inflates within tissue the surrounding tissue will stretch in the circumferential direction and compress in the radial direction. Assuming the tissue is reasonably homogeneous and isotropic before inflation then this will lead to anisoptropic stiffness in the region with increased stiffness in the circumferential direction and decreased stiffness in the radial direction. In an idealized case of shear wave propagation in a single direction across the entire domain we can predict the three dimensional pattern of increased and decreased stiffness bias that is reconstructed. This will lead to apparent softening in front of and behind the inclusion and a ring-like zone of increased stiffness around the inclusion, facing the wave. This is illustrated in Fig. 1. In practice, the shear wave direction will be more complex due to diffraction, reflection, superposition of multiple waves, etc., and therefore more complex stiffness patterns may be expected.

The method from Capilnasiu *et al*.^10^, used to extend the stiffness reconstruction method from Fovargue *et al*.^27^, is able to reconstruct the unbiased intrinsic stiffness of a deformed material given the macroscale deformation and nonlinear material law as input. This method is constructed in a mathematically rigorous manner using a perturbation analysis relating the two scales defined by ***v*** and ***u***, the small scale wave displacements and the large scale deformation, respectively. The analysis leads to the following set of equations governing the wave behaviour

3 $$\rho {\omega }^{2}{\boldsymbol{v}}+{\rm{\nabla }}\cdot ((\boldsymbol{\mathscr{G}}{\rm{{\prime} }}+i\boldsymbol{\mathscr{G}}{\rm{{\prime} }}{\rm{{\prime} }})\,:{\rm{\nabla }}{\boldsymbol{v}})+{\rm{\nabla }}p={\bf{0}}$$

4 $$\nabla \cdot {\boldsymbol{v}}=0$$

where $\boldsymbol{\mathscr{G}}{\rm{{\prime} }}$ and $\boldsymbol{\mathscr{G}}{\rm{{\prime} }}{\rm{{\prime} }}$ are fourth order tensors given by

5 $${\boldsymbol{\mathscr{G}}}_{ijml}^{{\rm{{\prime} }}}=\frac{1}{J}{[{\boldsymbol{F}}{{\rm{\nabla }}}_{{\boldsymbol{F}}}({{\boldsymbol{S}}}_{e}+{{\boldsymbol{S}}}_{p})+\boldsymbol{\mathscr{I}}{\boldsymbol{S}}]}_{ismn}{F}_{lm}{F}_{js}$$

6 $${\boldsymbol{\mathscr{G}}}_{ijml}^{{\rm{{\prime} }}{\rm{{\prime} }}}=\frac{\omega }{J}{({\boldsymbol{F}}{{\rm{\nabla }}}_{{\boldsymbol{F}}}{{\boldsymbol{S}}}_{v})}_{ismn}{F}_{lm}{F}_{js},$$

taken from Capilnasiu *et al*.^10^ assuming the fractional derivative parameter is 1. Further, ${{\mathscr{I}}}_{ijkl}={\delta }_{ik}{\delta }_{jl}$, ***F*** = ∇ ***u*** + ***I*** is the deformation tensor, $J=\,{\rm{\det }}\,\left({\boldsymbol{F}}\right)$, and ***S*** is the second Piola-Kirchoff (PK2) stress tensor and is the sum of elastic, ***S***_*e*_, viscous, ***S***_*v*_, and hydrostatic, ***S***_*p*_, parts. Depending on the form of ***S***, different approaches to reconstruction may be considered. If we know the deformation that caused the apparent anisotropy and hence biases, then this approach may be used to reconstruct the unbiased intrinsic stiffness of the material, thereby *undoing* the effect of the deformation.

Here, a neo-Hookean material law is assumed, giving the following strain energy function

7 $${W}_{e}(P,{\boldsymbol{F}})=\frac{{\mu }_{e}}{2}({I}_{\bar{{\boldsymbol{C}}}}-3)$$

where ${I}_{\bar{{\boldsymbol{C}}}}=\bar{{\boldsymbol{C}}}\,:{\boldsymbol{I}}$ is the first invariant of the isochoric version of the right Cauchy-Green deformation tensor, $\bar{{\boldsymbol{C}}}={J}^{-2/3}{\boldsymbol{C}}$, and *μ*_*e*_ is the intrinsic shear modulus of the material in the absence of macroscale deformation. This gives the elastic PK2 stress tensor

8 $${{\boldsymbol{S}}}_{e}=\frac{{\mu }_{e}}{{J}^{2/3}}\left({\boldsymbol{I}}-\frac{{I}_{{\boldsymbol{C}}}}{3}{{\boldsymbol{C}}}^{-1}\right).$$

This is inserted into Eq. (5) giving the formula for $\boldsymbol{\mathscr{G}}{\rm{{\prime} }}$

9 $${\boldsymbol{\mathscr{G}}}_{ikml}^{{\rm{{\prime} }}}=\frac{1}{J}({F}_{iq}{({{\rm{\nabla }}}_{{\boldsymbol{F}}}{{\boldsymbol{S}}}_{e})}_{qjmn}{F}_{ln}{F}_{kj}+{({{\boldsymbol{S}}}_{e})}_{nj}{\delta }_{im}{F}_{ln}{F}_{kj})$$

where the hydrostatic component of ***S*** is considered to be negligible and ∇_***F***_***S***_*e*_ is

10 $${\left({\nabla }_{{\boldsymbol{F}}}{{\boldsymbol{S}}}_{e}\right)}_{qjmn}=\frac{{\mu }_{e}}{{J}^{2/3}}\left(-\frac{2}{3}{\delta }_{qj}{F}_{nm}^{-1}+\frac{2}{9}{F}_{nm}^{-1}{C}_{qj}^{-1}{I}_{{\boldsymbol{C}}}+\frac{2}{3}{F}_{mn}{C}_{qj}^{-1}+\frac{2}{3}{I}_{{\boldsymbol{C}}}\left({F}_{qm}^{-1}{C}_{nj}^{-1}+{F}_{jm}^{-1}{C}_{qn}^{-1}\right)\right).$$

Since *μ*_*e*_ is a constant multiple of all terms in $\boldsymbol{\mathscr{G}}{\rm{{\prime} }}$ we define the following

11 $$\boldsymbol{\mathscr{G}}{\rm{{\prime} }}={\mu }_{e}\boldsymbol{\mathscr{H}}{\rm{{\prime} }}.$$

It is assumed that the relative effect on *μ*_*e*_ is the same as on the frequency dependent $G{\rm{{\prime} }}$, and so *μ*_*e*_ is simply replaced with $G{\rm{{\prime} }}$ in the relevant equations.

In order to maintain features of the reconstruction method that contribute to increased robustness we must solve for a scalar (complex-valued) stiffness. If the waves are processed differently for the elastic and viscous parts of the stiffness then these features are lost. Therefore we choose to scale the waves corresponding to the viscosity in the same way as the waves corresponding to the elasticity. Changes to viscosity due to deformation is outside the scope of this article and further we assume that any effect on the elasticity due to this choice is negligible. So, the equation which the reconstruction solves is then

12 $$\rho {\omega }^{2}{\boldsymbol{v}}+{\rm{\nabla }}\cdot ((G{\rm{{\prime} }}+iG{\rm{{\prime} }}{\rm{{\prime} }})(\boldsymbol{\mathscr{H}}{\rm{{\prime} }}:{\rm{\nabla }}{\boldsymbol{v}}))+{\rm{\nabla }}p={\bf{0}}$$

which is solved exactly as in Fovargue *et al*.^27^ only with $\boldsymbol{\mathscr{H}}{\rm{{\prime} }}:{\rm{\nabla }}{\boldsymbol{v}}$ replacing ∇ ***v***.

As opposed to Capilnasiu *et al*.^10^, the deformation field considered here is spatially variable, and so the value of ∇ ***v*** at each voxel is modified by the corresponding value of $\boldsymbol{\mathscr{H}}{\rm{{\prime} }}$ at that spatial location as a preprocessing step. If this reconstruction is run while assuming only the trivial deformation field, ***F*** = ***I***, but in the presence of actual deformation then the result will match the standard version of the reconstruction and will show biases due to deformation. However, if the correct deformation field is inserted then the reconstruction will recover the intrinsic unbiased stiffness, thereby undoing the effect of the deformation (in our case due to the pressurized inclusion).

### Deformation scaling

Using the stiffness reconstruction described above, we wish to find a macroscale deformation field which mimics the effect of the pressurized inclusion on the surrounding tissue. Our assumptions are that the surrounding tissue is nearly homogeneous and that the deformation introduces apparent heterogeneity in stiffness due to nonlinear effects. Therefore the deformation which most reduces this heterogeneity will be considered to be the correct deformation. To limit the possible choices we only consider scalings of a spherical deformation. The assumption is further justified in that most tumours grow in an approximately spherical way and that as objects inflate they tend towards a more spherical shape.

The family of deformation fields considered here are defined by an incompressible thick spherical shell model^33^, where *R*_0_ is the radius of the uninflated tumour and *r*_0_ is the radius of the tumour after inflation. In other words, *r*_0_ represents the real size of the tumour, whereas *R*_0_ represents the theoretical size of the tumour if all fluid and solid pressure were removed. We define a radial variable, *r*, corresponding to the inflated state, and a radial variable, *R*, corresponding to the uninflated state. Incompressibility then implies

13 $${r}^{3}-{R}^{3}={r}_{0}^{3}-{R}_{0}^{3}.$$

We define a scaling parameter for the deformation field, *α*, so that *α* = 0 corresponds to no inflation (or zero pressure) and *α* = 1 corresponds to inflation from a point,

14 $${R}_{0}=\left(1-\alpha \right){r}_{0}.$$

Moreover, *α* < 0 would imply a negative pressure, or pulling of instead of pushing on the surrounding tissue. Knowing *r*_0_ and the center of the tumour, ***c***_0_, and assuming a value of *α* allows the construction of the deformation field. At a point ***x*** that is a distance *r* from ***c***_0_, the magnitude of the radial deformation field is given by

15 $$\Delta r=r-R=r-3\sqrt{{r}^{3}-{r}_{0}^{3}+{\left(1-\alpha \right)}^{3}{r}_{0}^{3}}$$

and the direction is simply ***x***−***c***_0_. Scalings of this deformation field can be constructed by varying the value of *α*. Considering the example shown in Fig. 1, we would expect to see the described stiffness biases when *α* = 0 and an elimination of the biases when *α* = *α*_true_. Furthermore, *α* < 0 would lead to an increase in the magnitude of the biases and *α* > *α*_true_ to an inversion of the biases.

The correct scaling of *α* is considered to be the one that most reduces the heterogeneity of stiffness in the surrounding tissue. This is measured from the standard deviation of the reconstructed stiffness, $G{\rm{{\prime} }}$, in a spherical shell region of interest (ROI) surrounding the inclusion. The inner radius of the ROI is defined as being 3 pixels greater than *r*_0_. This is to balance being as close as possible to the inclusion but avoiding including $G{\rm{{\prime} }}$ values that have been affected by the deformation scaling within the inclusion and the inclusion edge itself, as the radius of the reconstruction window is approximately 3 pixels. The width of the ROI is chosen to be 4 pixels wide to include many pixels for robustness but avoid including pixels that have negligible change with varying deformation scalings. The *α* value which minimizes the standard deviation of $G{\rm{{\prime} }}$ is found by sweeping over potential values from -0.2 to 1 in steps of 0.01. To save some computational effort, stiffness reconstruction is limited to a bounding box surrounding the ROI.

### Pressure calculation

From *α* we use an analytic model of an inflating sphere to calculate the pressure. Equilibrium equations, and assuming a neo-Hookean material law, give the following for determining the radial stress^33^

16 $$\frac{\partial {\sigma }_{rr}}{\partial r}=2{\mu }_{e}\frac{{r}^{6}-{R}^{6}}{{r}^{5}{R}^{2}}$$

where, as before, we have the relations

17 $${R}^{3}={r}^{3}+{R}_{0}^{3}-{r}_{0}^{3}={r}^{3}+{\left(1-\alpha \right)}^{3}{r}_{0}^{3}-{r}_{0}^{3}$$

Integrating gives a formula for the radial stress

18 $${\sigma }_{rr}\left(r;\alpha \right)=2{\mu }_{e}{\int }_{{r}_{0}}^{r}\frac{{r}^{6}-{R}^{6}}{{r}^{5}{R}^{2}}\,{\rm{d}}r-{p}_{{\rm{inc}}}$$

where *p*_inc_ is the pressure in the inclusion. Noting that $R=R\left(r;\alpha \right)$, the pressure is found in terms of *α* then by

19 $${p}_{{\rm{inc}}}\left(\alpha \right)=2{\mu }_{e}{\int }_{{r}_{0}}^{\infty }\frac{{r}^{6}-{R}^{6}}{{r}^{5}{R}^{2}}\,{\rm{d}}r=2{\mu }_{e}{{\rm{lim}}}_{\rho \to \infty }{\left[\frac{{R}^{4}+4R{r}^{3}}{4{r}^{4}}\right]}_{{r}_{0}}^{\rho }=2{\mu }_{e}\left(\frac{5}{4}-\frac{{\left(1-\alpha \right)}^{4}+4\left(1-\alpha \right)}{4}\right)$$

**Figure 2:**
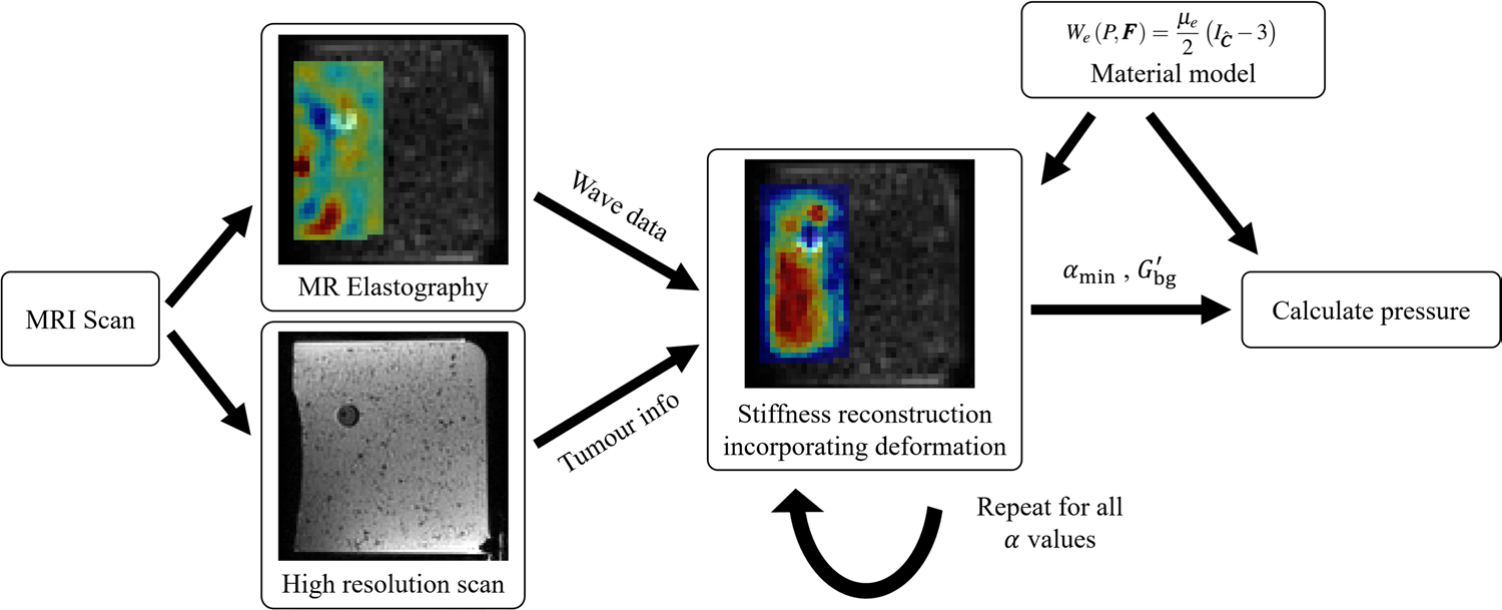
Flowchart showing an overview of the pressure reconstruction process. From an MRI experiment, both elastography data and some higher resolution data is acquired. These provide wave data for the reconstruction and information about the size and location of the tumour, respectively. A stiffness reconstruction is performed with wave data modified in accordance with a nonlinear material model and a deformation field defined by *α*. This reconstruction is repeated for all desired values of *α*. The *α* value found to minimize the stiffness biases is passed to the pressure calculation which is again based on the material model. The reconstruction may also be used to inform the pressure calculation of the tissue background stiffness, ${G}_{{\rm{b}}{\rm{g}}}^{{\rm{{\prime} }}}$.

The pressure is then dependent on only the background stiffness, *μ*_*e*_, and the value of *α* found by the method described above. As this portion of the method does not rely on frequency, it is appropriate to use the intrinsic *μ*_*e*_ of the material and not $G{\rm{{\prime} }}$. The stiffness reconstruction, deformation scaling, and pressure calculation are all implemented in MATLAB (Mathworks, Natick, MA, USA). An overview of the method is provided as a flowchart in Fig. 2.

### Simulations

Computational simulations are used to verify and test the method. These consist of two main parts: (1) a nonlinear solid mechanics simulation governing the expansion of a pressurized inclusion and (2) a linear wave simulation in the same domain to model the vibration during elastography. Both simulations are executed in CHeart, a finite element software specializing in the multi-physics of fluid and solid mechanics^34^.

Three inclusion shapes are modelled: a sphere, a bumpy inclusion, and an oblique egg-shaped inclusion. The inclusions (modelled as voids) all begin the inflation simulation at approximately 4 mm radius and are situated in the center of a cube domain with edge length 50 mm. Over the course of 60 load steps, the internal pressure of the inclusion is incrementally increased from 0 to 2.5 kPa. The higher end of this spectrum should be approximately in the range of total tumour pressure for real tumours^2,35,36^. Several additional spherical inclusions of varying initial size (3 mm, 5 mm, and 6 mm) are also modelled. This group of simulations was limited to 50 load steps (2.08 kPa) as the larger radii subject to the highest pressures led to problematic levels of deformation of the computational mesh. For all simulations, the domain is modelled as a neo-Hookean solid with *μ*_*e*_ = 1.5 kPa and with several boundary nodes fixed in place to restrain the simulation. The background stiffness is chosen to be near that of some real tissue, specifically breast (0.87 kPa^37^), brain (1.2–2.1 kPa^38^), and liver (2.0 kPa^39^), as these organs may be the most likely application of the method in future work. The finite element meshes are composed of approximately 210,000 to 230,000 tetrahedral ${{\mathbb{P}}}^{2}$-${{\mathbb{P}}}^{1}$ elements depending on the inclusion type and size. The inflation simulations took approximately 9 hours each to run on an HPC machine utilising 64 cores (Intel Xeon 2.67 GHz).

**Figure 3:**
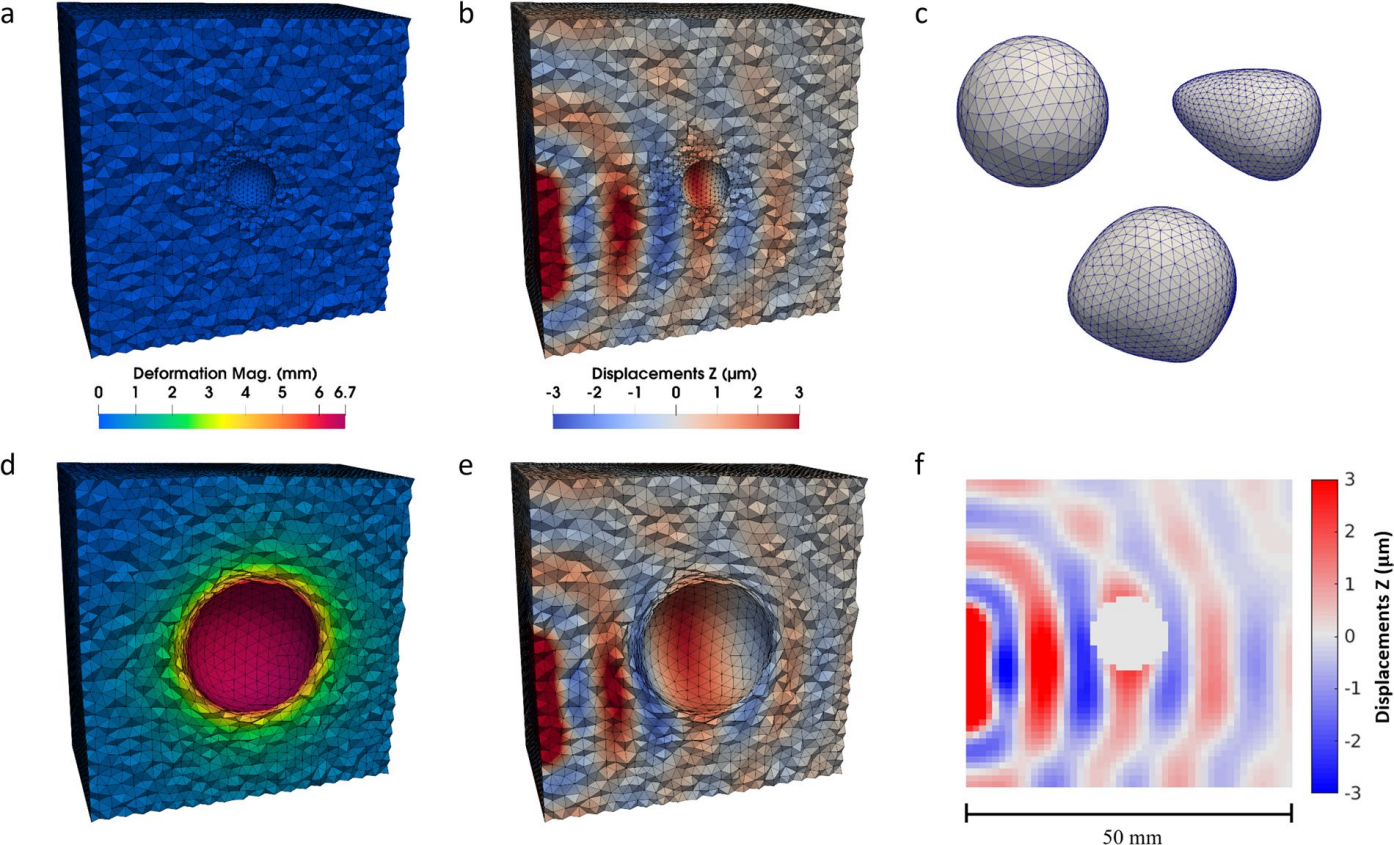
Overview of numerical simulations. (**a**,**b**) Show the mesh (cut in half) at time point 0 and display the deformation magnitude and real part of the z-displacements of the wave data, respectively. (**c**) Shows the three inclusions. (**d**,**e**) Show again the deformation and waves but at the final load step, 60. (**f**) Shows the same component of the wave data at load step 40 extracted onto an image.

The steady-state wave simulations are run on the same meshes as used for the inflation simulations. A portion of the boundary has Dirichlet type boundary conditions set to induce waves, where all displacement components are initialized. The wave frequency is 100 Hz and *G*^∗^ = 1.5 + *i*0.2 kPa. These simulations took approximately 8 hours each using 64 cores on the same HPC machine. The result of the wave simulation is extracted from the well-resolved unstructured finite element mesh (in the configuration from the desired inflation load step) onto a coarser uniform grid to represent a typical image formed from MR elastography (1 mm isotropic resolution). Figure 3 presents an overview of the different aspects of the simulations.

### Phantom experiments

A set of three phantom experiments were performed to further validate the method, using an expanding balloon catheter to model a pressurized tumour. The balloon catheter was inserted into an approximately cube-shaped 8 cm plastisol phantom (a different phantom was made for each experiment). Five MRE scans were performed at 210 Hz for each phantom, with 2 mm isotropic resolution. These five scans corresponded to five states: one uninflated state and with 100, 200, 300, and 400 *μ*L of water added into the balloon. Higher resolution images, used to estimate the inclusion position and radii, were also acquired in each state with 0.89 mm isotropic resolution. The higher vibrational frequency used here of 210 Hz, compared to human applications (~50 Hz), led to significant attenuation of the waves away from the transducer and balloon. Therefore, all processing of the wave data was limited to approximately the half of the phantom closer to the transducer to ensure correct alignment and orientation of the data, as lack of wave data can disrupt these calculations.

The fluid pressure was measured at each inflated state during the MRE experiments. As the balloon itself also contributes to the pressure, the fluid pressure was measured outside of the phantom as well (again at each state and after the MRE experiments) and these values were subtracted from the total fluid pressure to give only the pressure due to the restriction from the phantom. Replicate measurements were also made again, both inside and outside the phantom, but not during scanning. Mean measured pressure and radii values for each inflation state are included in Table 1. Finally, the intrinsic stiffness is found by fitting a neo-Hookean law to the measured pressure versus inflation volume data to give *μ*_*e*_ = 11.2 kPa. While the frequency, inclusion pressure, and background stiffness are all higher than would be expected *in vivo*, their relative contributions mimic tissue.

**Table 1:** Reconstructed phantom pressure results for all inflation levels and for all three experiments. Mean reconstructed pressure, mean measured pressure, and mean measured radii are also listed with standard deviations.

Inflation (*μ*L)	Meas. Radius (mm)	Meas. Pres. (kPa)	Recon. Pres. (kPa)			
	Mean ± SD	Mean ± SD	Exp 1	Exp 2	Exp 3	Mean ± SD
0	1.66 ± 0.11	0	2.57	1.31	12.72	5.54 ± 6.26
100	1.90 ± 0.15	6.31 ± 2.18	3.38	4.55	14.37	7.44 ± 6.04
200	2.58 ± 0.32	15.02 ± 4.07	22.61	23.74	22.15	22.83 ± 0.82
300	3.51 ± 0.12	19.74 ± 3.27	21.92	27.10	23.96	24.32 ± 2.61
400	4.01 ± 0.13	22.63 ± 1.04	21.69	24.64	22.15	22.83 ± 1.58

## Results

### Verification of pressure calculation

**Figure 4:**
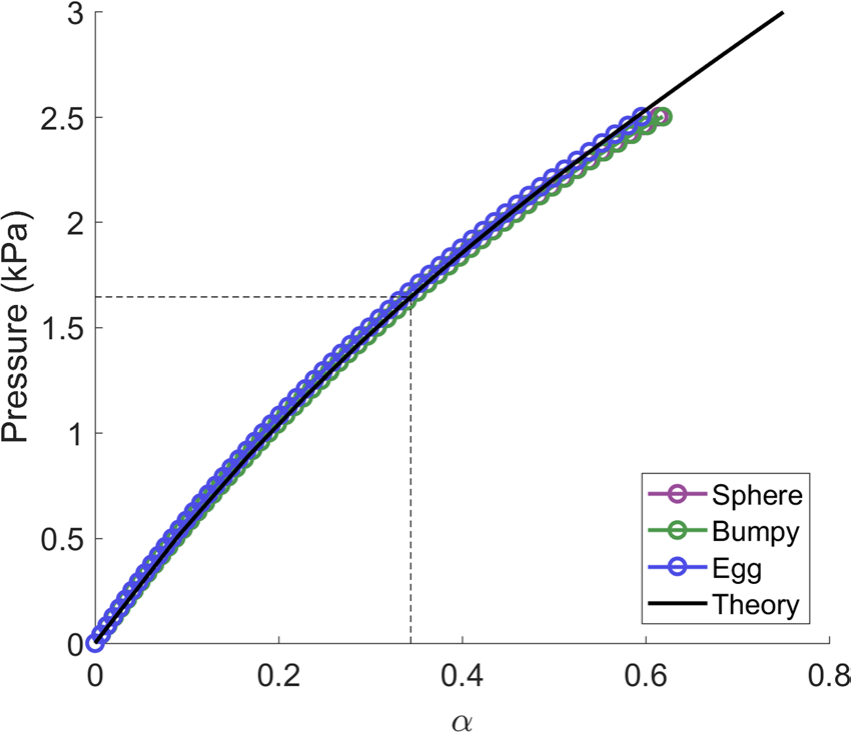
Comparison of *α*-pressure relationship defined in Eq. (19) (black line) to simulation results at every load step (purple, blue, and green circles). Also, as an example, dotted lines show the reconstructed *α* value (0.34) from the spherical data at load step 40 and the corresponding pressure value (1.63 kPa).

To verify the method for converting the deformation scaling parameter, *α*, to pressure, the relationship defined by Eq. (19) is compared to the pressure and *α* values from the simulations for the three inclusion shapes (Fig. 4). In the simulations the pressure defines the progress, that is, the pressure is increased uniformly every load step and the *α* is then calculated from the result. Specifically, *r*_0_ (at every load step) and *R*_0_ (from load step 0) are calculated as the average distance from the center of the mesh to all mesh nodes making up the inclusion surface. Then *α* is found from Eq. (14).

### Pressure values from simulation data

**Figure 5:**
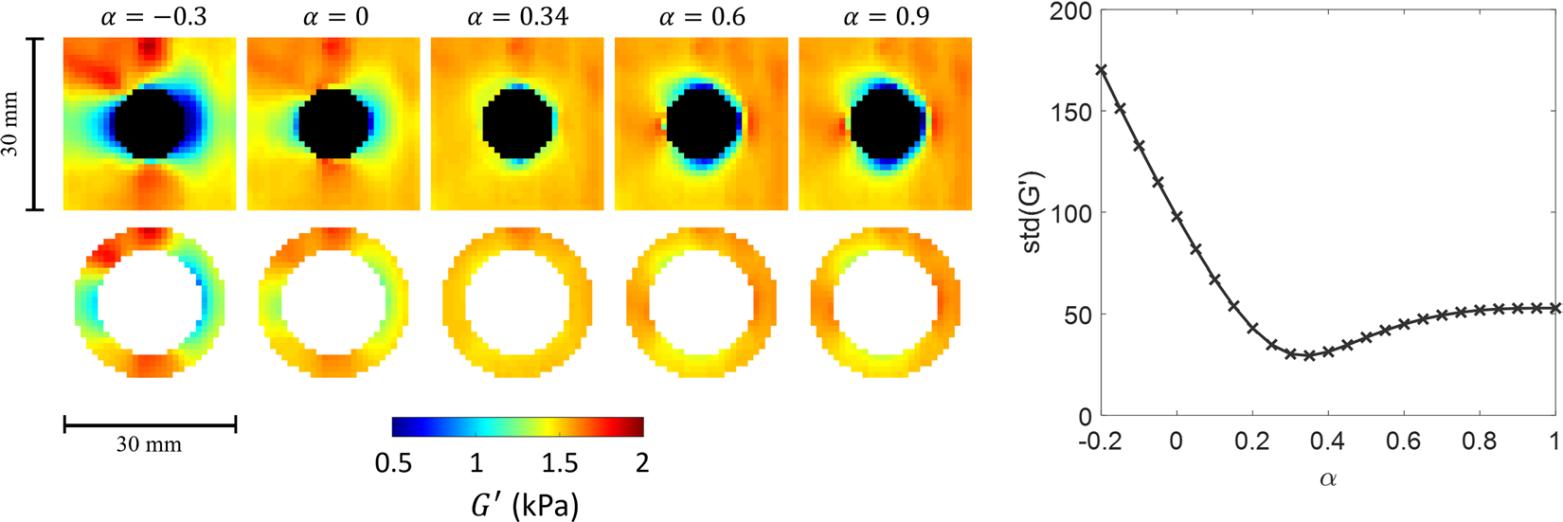
Overview of the reconstruction process using the spherical inclusion at load step 40 as an example. (Left, Top Row) Images of $G{\rm{{\prime} }}$ at selected values of *α*. Black pixels lie in the void of the inclusion. (Left, Bottom Row) The same images of $G{\rm{{\prime} }}$ but only showing the ROI used to compute the standard deviation. The expected bias is observed when *α* = 0, increased for *α* < 0, corrected at *α* = 0.34, and inverted for *α* > 0.34. (Right) A plot of the standard deviation of $G{\rm{{\prime} }}$ versus *α* values. For this illustrative example, *α* was incremented by 0.05 (whereas for reported results *α* was incremented by 0.01).

Wave images were extracted at every 10 load steps corresponding to incremental pressure increases of 2.5/6 (≈0.417) kPa from 0 to 2.5 kPa for the simulations comparing shape (and 0 to 2.08 kPa for the simulations comparing initial radius). Deformation parameters, ***c***_0_ and *r*_0_, were determined from the location of mesh nodes on the inclusion surface. Pressure was reconstructed at each extracted time point for the inclusion types as described in the Methods section. Figure 5 shows stiffness reconstructions for various values of *α* for the spherical inclusion (initial radius 4 mm) at load step 40 (1.6667 kPa) at the center slice. The reconstructed stiffness in a cross section of the ROI is also shown. A plot of standard deviation of $G{\rm{{\prime} }}$ versus *α* is shown as well. Here, *α* is incremented by 0.05 for illustrative purposes, but for other results *α* was incremented by 0.01. The *α* and pressure calculated for this data set (0.34 and 1.63 kPa, respectively) are also indicated in Fig. 4.

**Figure 6:**
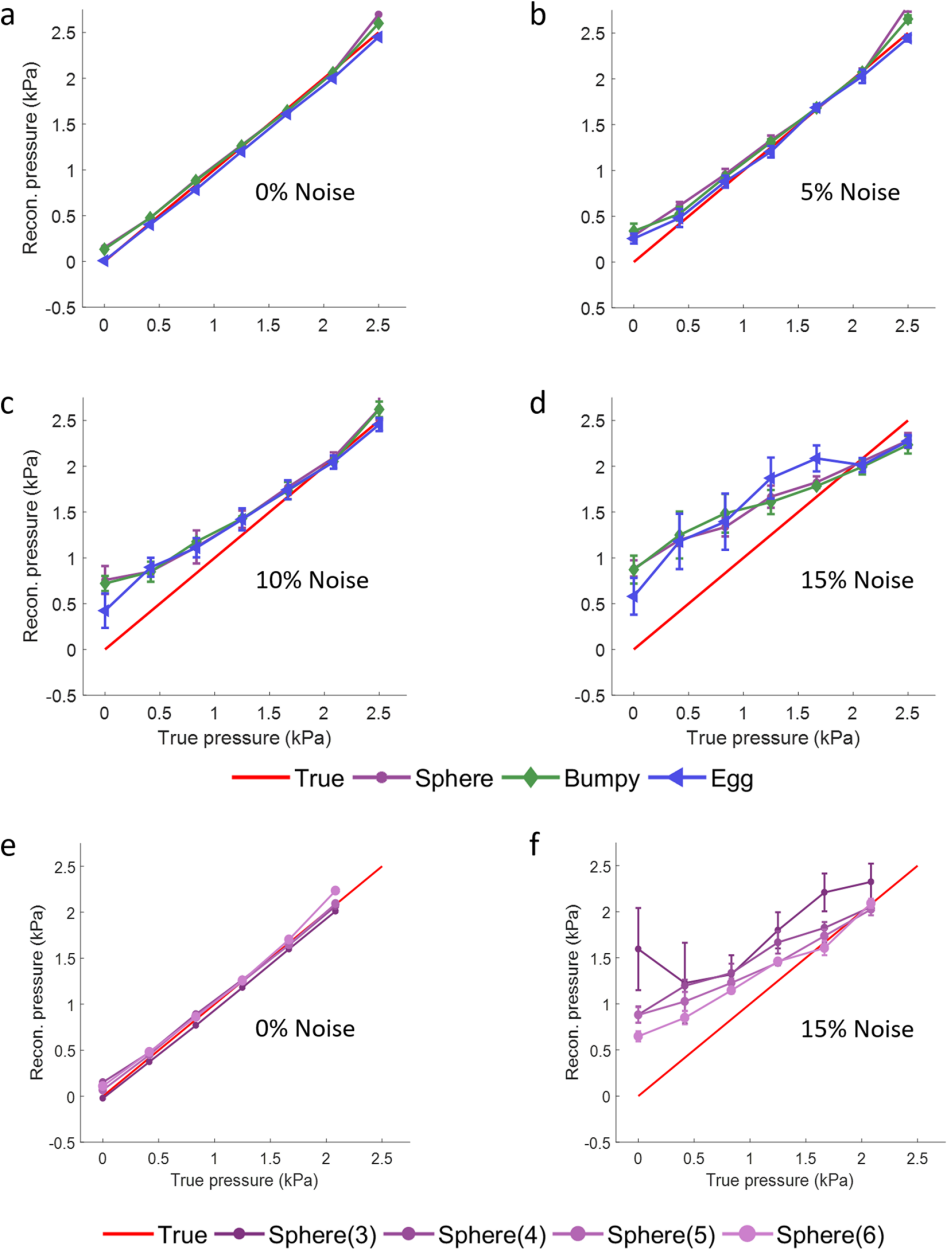
Reconstructed pressure value for each inclusion at every 10 load steps versus the true pressure from the simulation. (**a**–**d**) Show 0%, 5%, 10%, and 15% added noise, respectively, for the different shaped inclusions. (**e**,**f**) Show 0% and 15% noise for the different sized spherical inclusions (3 mm, 4 mm, 5 mm, 6 mm). Points show mean and error bars show standard deviation of reconstructed pressure over three runs.

Tests were also run on all data sets with added random noise on all six components of the wave data. The noise was drawn from a uniform distribution from [−*n*_0_, *n*_0_] where *n*_0_ was approximately 5%, 10%, and 15% of the maximum wave amplitude in the ROI for calculating stiffness variation. For cases with noise, Gaussian smoothing with 3 pixel support and *σ* = 1 pixel was applied after noise was added to mimic reality. Figure 6 displays the reconstructed pressure results of the noise-less and added-noise tests versus the true pressure. Each added-noise case was run three times with different random noise and the standard deviation of the resulting pressure is displayed as error bars with the mean of the three reconstructed pressure values. The average (over the three shapes) percent errors at the highest pressure level for the 4 noise cases (0%, 5%, 10%, 15%) are 4.6%, 6.8%, 3.9%, and 9.5%. For the differently sized spheres at 0% and 15% noise, the averaged percent errors are 3.0% and 4.1%.

### Pressure values from phantom data

Pressure was reconstructed for all five inflation states for each of the three experiments. The same Gaussian smoothing was applied to the wave data as for the simulations. The stiffness variation ROI was constructed in the same manner, however, depending on the inflation level there was not always enough slices to cover the entire spherical shell in the slice direction, so as many as possible were used. For the phantom data, an additional step of smoothing the reconstructed stiffness was also applied, so that the variance calculation was more influenced by larger scale variation due to the inflation and less by small scale variation often seen in experimental data.

**Figure 7:**
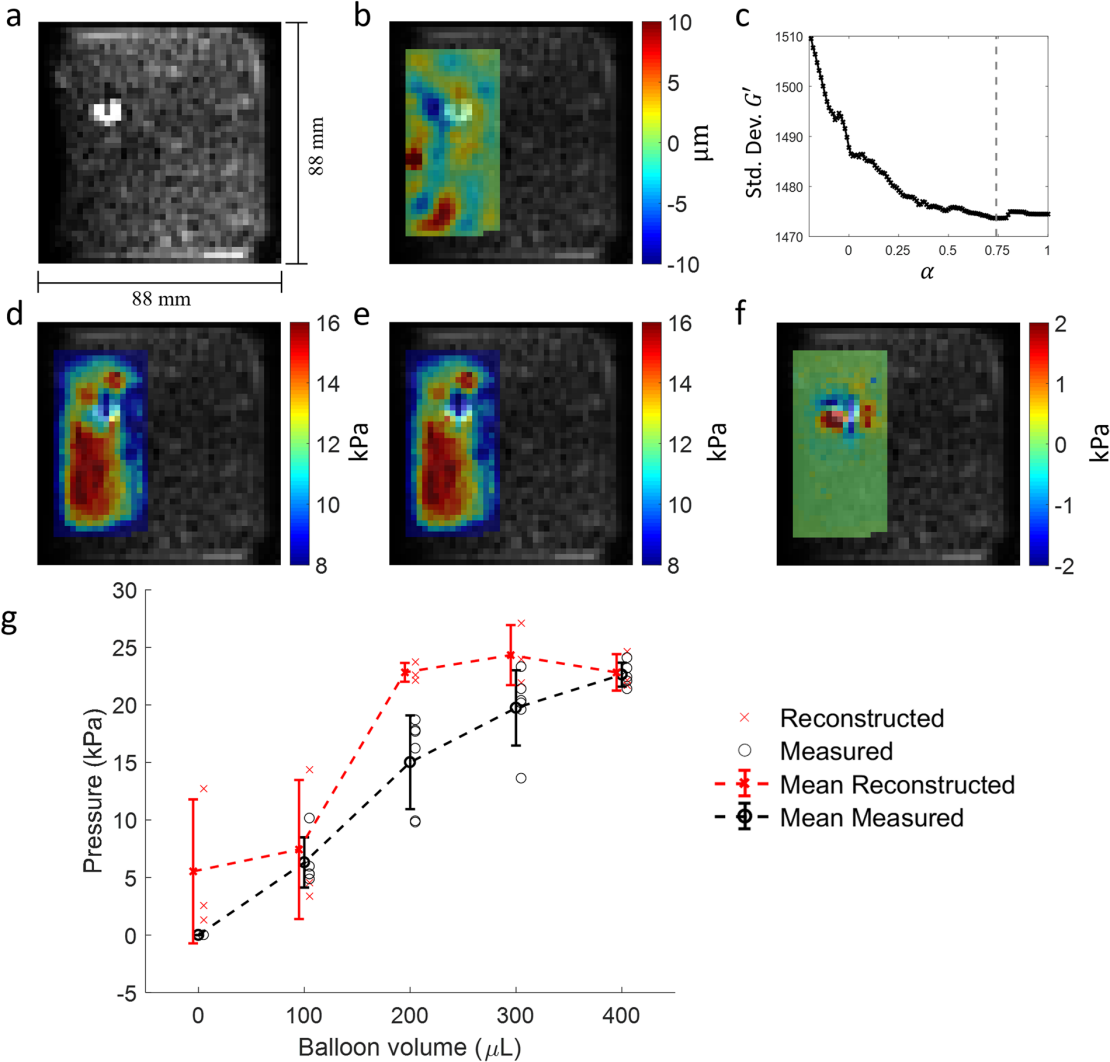
Phantom experiments and overall results. (**a**) MRE-resolution magnitude image of one phantom with catheter balloon and maximum 400 *μ*L inflation. (**b**) Example component of wave data, ${\rm{Re}}\left({u}_{x}\right)$, overlaid on the same magnitude image. (**c**) Standard deviation of $G{\rm{{\prime} }}$ versus selected *α* values with a vertical dotted line indicating the minimum. (**d**) $G{\rm{{\prime} }}$ at *α* = 0 overlaid on the same magnitude image. (**e**) $G{\rm{{\prime} }}$ at *α* = *α*_min_ = 0.72. (**f**) The difference between $G{\rm{{\prime} }}$ at *α*_min_ and $G{\rm{{\prime} }}$ at *α* = 0 showing, as expected, the negative of the hypothesized stiffness bias pattern. (**g**) Compiled pressure results for all phantom experiments and inflation levels. Individual reconstructed and measured pressures are presented as singular points. Mean reconstructed and measured pressures are plotted with standard deviation as error bars.

Figure 7 shows example wave and reconstructed stiffness for a data set at 400 *μ*L inflation. All elastogram images from the phantom experiments are included in Supplementary Material. Figure 7 also displays the reconstructed pressure and measured pressure at each inflation level. Individual values are displayed along with the mean and standard deviation. For the 0 inflation data, pressure was not measured but assumed to be 0. At each inflated state, six measurements of pressure values were recorded as there were three overall experiments and two pressure measurements (one during scanning and one replicate afterwards). However, one replicate measurement for the 400 *μ*L is missing as the balloon popped and one measurement for 100 *μ*L was discarded as post-analysis revealed the initially recorded pressure was not stable over time potentially indicating a leak. Table 1 includes all reconstructed pressure values as well as means and standard deviations for measured pressures, reconstructed pressures, and mean radius for each inflation level. The percent errors between measured and reconstructed pressure for the four non-zero inflation cases are 17.9%, 52%, 23.2%, and 0.9%. The absolute errors between mean measured and reconstructed pressures for all 5 cases are 5.5, 1.1, 7.8, 4.6, and 0.2 kPa. The largest three inflation levels are not able to be well distinguished from one another, however they do show significantly higher pressure than the 0 *μ*L case (p = 0.009, 0.0086, 0.0097). The 100 *μ*L case is not significantly different from the 0 *μ*L case (p = 0.72).

## Discussion

Good agreement is seen for the *α*-pressure relationship (Eq. (19)) as displayed in Fig. 4. This is expected for the spherical inclusion as the theory is based on the inflation of a sphere, however both the bumpy and egg inclusions also match well with the analytic pressure which helps to validate this choice of model. For lower values of *α* the egg shape pressure values are slightly overestimated, presumably because it is the furthest from a spherical shape. As *α* becomes larger, the sphere and bumpy pressures begin to diverge from theory. This may be due to a violation of the assumption of the inclusion being in an infinite medium (i.e. the simulation boundaries become significant for the largest of inflations). This presumably affects the egg shape as well, however, as it is overestimated for small *α* it then appears to become closer for larger *α*.

Pressure reconstructions from the numerical examples match the true pressure very well for zero and low noise cases and for all inclusion shapes and sizes as seen in Fig. 6. This again helps to validate the choice of a spherical model even for cases which diverge from that shape. The error at the highest pressure (final load step), evident in the 0%, 5%, and 10% cases, may again derive from the simulation boundaries becoming significant. For higher noise cases there begins to be divergence but the pressure values are still reasonably accurate for higher pressures. Percent errors for the highest two pressure levels remain near or under 10% for all shapes and sizes. Overall, as noise increases, the variation of reconstructed pressure increases (more so for lower values) and the accuracy of lower pressure values decrease. Noise in the wave data will lead to variation (or false heterogeneity) in the stiffness result. Therefore, for small pressures values, the true change in stiffness due to deformation may be hidden by variation due to noise and only for larger pressures is the deformation variation able to be robustly seen and minimized by choice of *α*. Also, the magnitude of the deformation is dependent on radius, and so small radii will have less effect on stiffness which may lead to a stronger dependency on the homogeneity assumption and therefore more variability in the resulting pressure. This is clear too, in the results for the spherical inclusion with the smallest radius as it clearly suffers the most with added noise. We also note an overall tendency for the pressure to be overestimated for these smaller radii inclusions in the presence of noise. The background stiffness and tumour pressure range of the simulations were chosen to be in the expected ranges of real tissue and tumours. There is a scaling relationship between tumour pressure and tissue stiffness, in that for the same amount of strain (deformation of the tumour), the pressure and tissue stiffness will positively correlate, and so the simulations inherently cover a wider range of values at least in terms of recovering pressure.

As seen in Fig. 7, the behaviour of the reconstructed pressures from the phantom experiments match with measured pressures. The reconstructed pressures follow a general increase with inflation level and pressures are of the correct magnitude. Reconstructed pressures at the larger inflation levels are significantly higher than from the zero pressure state. The lower inflation levels also show more variation, consistent with the simulation results. Overall the phantom data is especially promising considering several factors contribute to the data being more difficult to process than simulation. Besides some inherent noise during imaging, there may also be some intrinsic stiffness heterogeneity in the undeformed phantom, imaging errors in the wave data, or other model-data mismatch. The phantom also has a hole running through the middle of the inflating sphere for the catheter that could affect wave propagation (and therefore stiffness) and/or the inflation of the sphere. Finally, the inclusion is smaller relative to voxel size than for the simulation. As explained above, and as seen in the simulation result for the smallest spherical inclusion, this could also lead to more variability. While it may be promising overall that pressures from these smaller inclusions are reasonably reconstructed, the sizes are small compared to real tumours which does represent a limitation of the experiment as a whole. During the preliminary design of the experiment it became clear that 400 *μ*L inflation was the largest inflation where we could guarantee that the phantom material would not tear.

The computation of pressure is inexpensive as the underlying stiffness reconstruction utilises a local moving window and a direct least squares solve^27^, although there is a slight increase in time over the original method due to re-scaling the wave derivatives. Stiffness reconstructions using this method typically take on the order of 10s of seconds, simply scaling with the number of voxels in the data set. Likewise, the computational effort of the pressure reconstruction scales with the number of desired *α* values. For example, the pressure reconstruction in its current MATLAB implementation for a phantom data set takes about 15 minutes if *α* is incremented by 0.01 from −0.2 to 1. In this work, the stiffness reconstruction was typically restricted to a bounding box surrounding the ROI, however this could be further restricted to the exact voxels comprising the ROI. Moving forward, the time can be substantially and straightforwardly reduced, if need be, as the sweep of *α* values is trivially parallelizable.

Generally speaking, future *in vivo* pressure data will be expected to be more difficult to estimate, coming from factors such as stiffness variability due to noise, inherent heterogeneity, or data-model mismatch. Robustness of the method could be increased by collecting more data through typical routes to improve wave image quality or by collecting multiple data sets with differing wave sources. The latter could then require the *α* reconstruction process to find the *α* which minimizes the stiffness variance of multiple data sets, as these would have different *k*-vector directions and therefore different stiffness biases surrounding the tumour. For now, we assume any potential future *in vivo* data would conform well to the assumptions that the tumour is nearly spherical in shape, well-embedded but clearly delineated from surrounding tissue, and that accurate values for the tumour center and radius can be found. Understanding the effects of straying from these assumptions is left for future work. While the volume of tissue affected by the deformation is small, relative to the whole organ for example, MRE should be able to accurately reconstruct stiffness values, at least for approximately the ratios of tumour size, deformation magnitude, wavelength, noise, and pixel size described here. Previous work has shown accurate stiffness reconstructions of small inclusions of a similar size (in terms of pixels per wavelength) as the affected area here^40–42^.

Possible future *in vivo* studies could be better suited in two ways however. First, *in vivo* data will not have the same catheter hole, as the phantom does, and so the tumour will be more properly embedded in the tissue. Second, the tumour will support shear waves. The worst case here is represented by the simulations where the tumour is replaced with a void which creates a very sharp discontinuity in the wave data and affects the stiffness reconstruction near the boundary. Similarly, the balloon in the phantoms is filled with water and therefore does not support shear waves, creating a less noticeable but extant discontinuity. We also note that some version of this method may be applicable to ultrasound elastography, however it is only directly applicable to a 3D image stack of 3D displacement data.

This paper presented a proof-of-concept method to infer tumour pressure noninvasively using stiffness measurements from MRE and the application of nonlinear biomechanical models. Essentially, the pressure is found from undoing the presumed stiffness biases resulting from the large scale deformation caused by the outward push from the pressure. The method was tested on simulated pressurized inclusions with results matching well with true pressure when varying pressure level, inclusion shape, initial inclusion size, and added noise. Phantom experiments were run with catheter balloons inflated to multiple levels and pressure was reconstructed from the resulting MRE images. Here the mean pressure over several experiments generally matched the behaviour of the experimentally measured pressure. This method offers a potential way to measure an important biomarker noninvasively. Future work includes further validation via *ex vivo* and animal studies as well as application to *in vivo* patient data.

## Supplementary information

Supplementary Information.
