# Supplementary material for: Towards noninvasive estimation of tumour pressure by utilising MR elastography and nonlinear biomechanical models: a simulation and phantom study
Source: Sci Rep. 2020 Mar 27;10:5588. doi: 10.1038/s41598-020-62367-3 (PMC7101441; doi:10.1038/s41598-020-62367-3)
Supplement: Supplementary file 1 — Supplementary Information. [file 41598_2020_62367_MOESM1_ESM.pdf]

Supplementary material for “Towards noninvasive estimation of tumour pressure by utilising MR elastography and nonlinear biomechanical models: a simulation and phantom study”

Daniel Fovargue, Marco Fiorito, Adela Capilnasiu, David Nordsletten, Jack Lee, Ralph Sinkus

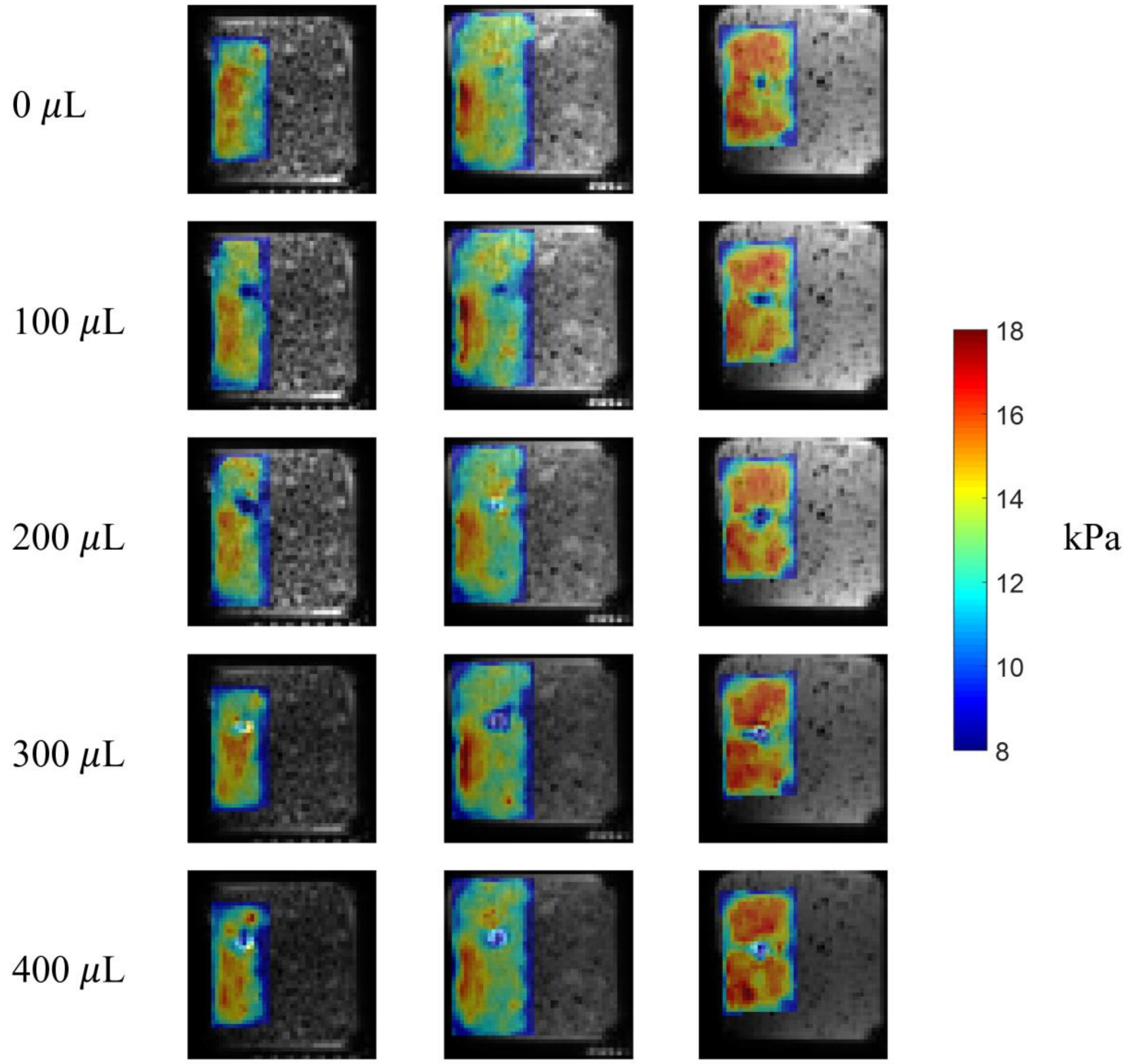

Figure S1. Elastograms from all fifteen phantom experiments overlaid on their respective MRE-resolution magnitude image. Each column displays one of the three experiments and the five rows display the increasing inflation levels. The elastograms show G' from the middle slice of the data without accounting for any deformation (or equivalently with $\alpha = 0$).
